# Middle mediastinal thymic carcinoma with cystic findings in radiologic images: a case report

**DOI:** 10.1186/s40792-016-0254-2

**Published:** 2016-11-07

**Authors:** Shuichi Shinohara, Koji Kuroda, Taiji Kuwata, Masaru Takenaka, Soichi Oka, Yasuhiro Chikaishi, Ayako Hirai, Naoko Imanishi, Fumihiro Tanaka

**Affiliations:** Second Department of Surgery, School of Medicine, University of Occupational and Environmental Health, 1-1 Iseigaoka, Yahatanishi-ku, Kitakyushu, 807-8555 Japan

**Keywords:** Cyst, Middle mediastinum, MRI, Thymic carcinoma

## Abstract

**Background:**

Thymic carcinomas are usually detected in the anterior mediastinum. Thymic carcinoma occurring in the middle mediastinum and showing an image of a cyst is extremely rare.

**Case presentation:**

A 64-year-old man with a middle mediastinal tumor detected incidentally by a CT scan was referred. Chest CT showed an entirely cystic mass in the middle mediastinum between the bilateral brachiocephalic vein and trachea. We resected the tumor completely in the right recurrent nerve because it had invaded the nerve. Immunohistochemical features showed a thymic carcinoma pattern. The final diagnosis was thymic squamous cell carcinoma occurring in the middle mediastinum.

**Conclusion:**

To our best knowledge, this is the first report of a thymic carcinoma occurring in the middle mediastinum, as demonstrated by histopathological findings with immunohistochemical features.

## Background

Thymic neoplasms usually are detected in the anterior mediastinum. Fewer than 4% of thymic tumors occur in the middle mediastinum, defined as mediastinal space surrounded by the left brachiocephalic vein, superior vena cava, esophagus, trachea, and main bronchus up to the cross section of the left brachiocephalic vein and the center of the trachea by the Japanese Association for Research on the Thymus [1]. Thymic carcinoma is a rare thymic neoplasm. Moreover, there are few reports of middle mediastinal thymic carcinomas histopathologically diagnosed as having immunohistochemical features. We report a rare case of thymic carcinoma in the middle mediastinum that had cystic findings on computed tomography (CT) and magnetic resonance imaging (MRI).

## Case presentation

**Fig. 1:**
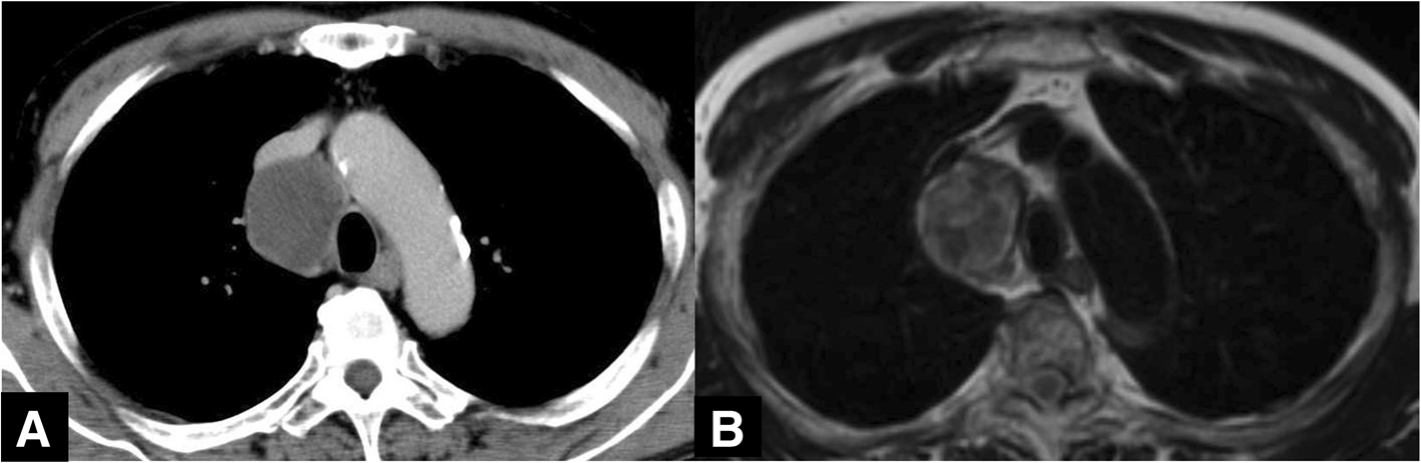
**a** Chest CT scan shows a low-density mass in the middle mediastinum surrounded by the brachiocephalic vein, brachiocephalic artery, and trachea. **b** T2-weighted MRI reveals the main tumor to have a heterogeneous isodense signal intensity, and the tumor was encapsulated by a low-signal area

A 64-year-old man was referred to our hospital for a middle mediastinal tumor detected incidentally by a CT scan. Chest CT showed an entirely cystic mass with a thick capsule slightly enhanced in the middle mediastinum between the bilateral brachiocephalic vein and trachea (Fig. 1a). At CT scan, the thymus is of normal size and located in the anterior mediastinum as a low-density triangle area. The mass had no solid component. T2-weighted MRI revealed that the main tumor had a heterogeneous isodense signal intensity and that the tumor was encapsulated by a low-signal area (Fig. 1b). There was no gadolinium-enhanced area in this tumor. This radiologic finding indicated the possibility of the mass being a hemorrhagic cyst, bronchogenic cyst, neurogenic tumor, or teratoma, with a small proportion of fat component.

**Fig. 2:**
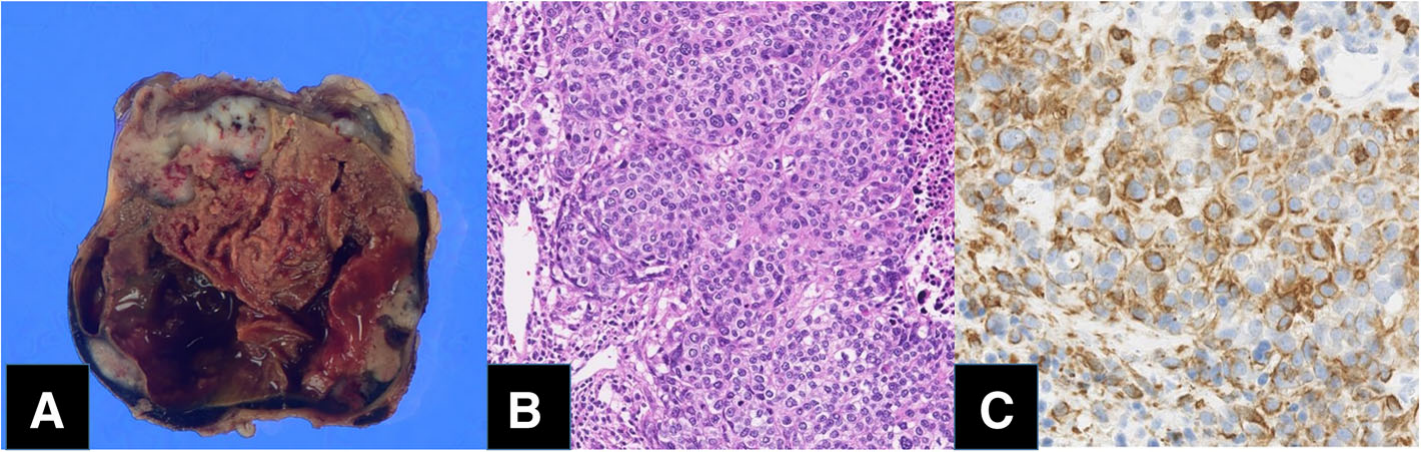
**a** Macroscopic findings of cross-sectional slices showed a small solid component (8 mm). **b** Microscopy revealed a proliferation of markedly atypical polygonal epithelial cells having hyperchromatic nuclei (×400). **c** Immunohistochemically, tumor cells are positive for CD5 (×400)

The patient was placed in the left lateral decubitus position, and a right thoracic approach with three-port video-assisted thoracoscopic surgery (VATS) was performed. The tumor was surrounded by the trachea, right main bronchus, brachiocephalic artery, superior vena cava (SVC), and left brachiocephalic vein. It was greatly adherent to the lateral trachea, right main bronchus, and recurrent nerve. Due to the complicated adhesiolysis in the anterior brachiocephalic artery and tumor involving the recurrent nerve, we decided to change the procedure to open thoracotomy. The tumor and recurrent nerve whose length was about 10 mm were removed while keeping them encapsulated. The tumor measured 45 × 35 × 30 mm and contained red–brown necrotic tissue surrounded by a fibrous capsule. Cross-sectional slices showed a small solid component (8 mm). The tumor nodule existed along a fibrous capsule multiply (Fig. 2a).

Microscopic findings showed a proliferation of markedly atypical polygonal epithelial cells having hyperchromatic nuclei associated with extensively necrotic and hemorrhagic areas (Fig. 2b, c). The recurrent nerve was involved with the carcinoma cells. Immunohistochemically, the carcinoma cells were positive for AE1/AE3, CAM5.2, p40, p63, CK34betaE12, CD5, and bcl-2 but negative for CK5/6, TTF-1, c-kit, AFP, and CD30. This feature indicated poorly differentiated, thymic squamous cell carcinoma in pathological T3N0M0 stage III. The margin of the resected tumor was free of disease.

Adjuvant concurrent chemoradiotherapy was performed. We administered monthly carboplatin plus paclitaxel for four courses, and the radiation therapy dose was 50 Gy. There was no recurrence 6 months after surgery.

## Conclusions

To our best knowledge, this is the first report of a thymic carcinoma occurring in the middle mediastinum, as demonstrated by histopathological findings with immunohistochemical features. Moreover, radiological findings demonstrating a cyst with no solid component in the middle mediastinum made preoperative diagnosis of a thymic carcinoma difficult.

Thymic carcinoma is an uncommon neoplasm and occurs in 5.5 % of all resected mediastinal tumors [2]. Furthermore, a middle mediastinal thymic carcinoma is extremely rare. Thymic carcinoma occurs in the anterior mediastinum, and ectopic thymic carcinomas, which are carcinomas that show thymus-like differentiation, are rare [3]. Ectopic thymic carcinoma is reported in cases of intrathyroid neoplasms [3], even though the first case reported was that of a middle mediastinal thymic carcinoma with histopathologic features. We think ectopic thymus tissue existed in middle mediastinum and it became a progressive neoplasm.

Thymic carcinoma is difficult to diagnose microscopically, whereas immunohistochemical features are greatly useful to make an exact diagnosis. CD5 is specific to the thymic epithelial cell, and metastatic tumor is excluded [4, 5]. To distinguish thymic squamous cell carcinoma from pulmonary squamous cell carcinoma is quite difficult. However, CD5 positive is useful for accurate diagnosis because pulmonary squamous cell carcinoma is consistently negative for CD5 by a large study of 1465 non-small cell lung cancer cases [5]. We diagnosed thymic squamous cell carcinoma by both microscopic findings and immunohistochemical findings. Middle mediastinal thymic carcinoma diagnosed by only microscopic findings was reported [6]. However, the immunohistochemical findings did not indicate thymic carcinoma because it was negative for CD5, which is specific to the thymic epithelial cell, and assessment for other thymic carcinoma markers (p53, c-kit, and bcl-2) was not performed. In our case, the immunohistochemical feature was coincident with thymic squamous cell carcinoma, and the possibility of other metastatic neoplasms in the mediastinum was excluded by imaging tests.

Mediastinal tumors showing cystic findings are generally benign. Malignancy has been reported in 2.4% of all cases with cystic findings detected by mediastinum CT [7]. Focal cystic change with thymic neoplasm, occurring in 40% of resected thymomas, is usual because thymic carcinomas and thymomas contain fluid, bleeding, and necrotic tissue with degeneration [8]. When high-grade degeneration developed, the tumor demonstrated cystic findings, such as in this case. Cystic thymomas have occasionally been reported, even though cystic thymic carcinoma is rare [8]. The malignant component in the microscopic findings was too small to be detected by imaging tests, and it made difficult to consider malignant tumor before operation.

Thymic carcinoma can occur in the middle mediastinum. Moreover, when cystic findings are noted in the middle mediastinum, thymic carcinoma should be considered in the differential diagnosis because the operative strategy and therapy of thymic carcinoma differ from those of other benign tumors.

## Abbreviations

CT: Computed tomography
MRI: Magnetic resonance imaging
